# Efficacy of sealants with surface pre-reacted glass (S-PRG) in retention, fissure penetration and occlusal caries Inhibition: Short title: efficacy of S-PRG sealants

**DOI:** 10.1007/s00784-025-06551-7

**Published:** 2025-10-13

**Authors:** Steve H.C. Yeh, Mohamed M.A. Abdalla, Phoebe P.Y. Lam, Cynthia K.Y. Yiu

**Affiliations:** 1 Paediatric Dentistry, Faculty of Dentistry, University of Hong Kong, Pokfulam, Hong Kong SAR Hong Kong; 2 Restorative Dental Sciences, Faculty of Dentistry, University of Hong Kong, Pokfulam, Hong Kong SAR Hong Kong

**Keywords:** S-PRG sealant, Fissure sealant, Penetration, Caries, Mineral density

## Abstract

**Objectives:**

This in vitro study aimed to evaluate the effect of different application protocols of surface pre-reacted glass-ionomer (S-PRG) sealant on penetration ability, retention, and occlusal caries inhibition, in comparison with resin-based sealant (RBS) under simulated oral conditions.

**Materials and methods:**

Eighty-four extracted human third molars were randomly assigned to five groups: S-PRG sealant applied per manufacturer’s instructions (G1), S-PRG with 37% phosphoric acid etching pretreatment (G2), S-PRG with fluoride varnish pretreatment (G3), RBS (G4), and a no-sealant control (G5). Sealant retention was assessed through visual inspection following water storage at 37 °C and thermocycling, whereas penetration depth was evaluated using scanning electron microscopy (SEM). Caries inhibition was evaluated via mineral density changes (ΔMDV) measured by micro-CT after 21-day pH cycling and *Streptococcus mutans* bacterial challenge. Statistical analysis included Kruskal-Wallis tests, Mann-Whitney U tests with Bonferroni correction, Chi-square tests, Spearman’s correlation, and multiple linear regression analyses.

**Results:**

No statistically significant differences in sealant retention were found among the groups. RBS (G4) exhibited the highest median penetration (100%), followed by G2 (85.0%), G1 (59.5%), and G3 (44.5%), with significant differences among specific pairwise comparisons (*p* < 0.001). No significant difference in ΔMDV were observed in pH cycling, but bacterial testing revealed significantly lower ΔMDV in G2 and G4 compared to G3 and G5 (*p* ≤ 0.005).

**Conclusions:**

S-PRG sealants pretreated with phosphoric acid etching demonstrated significantly greater caries inhibition effects compared to using self-etched primer alone, and its caries prevention efficacies were comparable to that of RBS.

**Clinical relevance:**

These in vitro findings support the use of phosphoric acid etching to enhance the performance of S-PRG sealants.

**Supplementary Information:**

The online version contains supplementary material available at 10.1007/s00784-025-06551-7.

## Introduction

Fissure sealants have gained substantial endorsement from established dental guidelines for their r [1, 2]. These preventive applications are especially recommended for occlusal surfaces with deep pits and fissures on molars, where plaque accumulation poses a t [2].

Resin-based sealant (RBS) is a common choice among dental practitioners, with ample evidence supporting its efficacy in prevention of occlusal caries in permanent molars. Studies with moderate certainty of evidence have shown RBS’s superior efficacy compared to no intervention [3]. However, RBS have some limitations, including moisture sensitivity, the need for meticulous procedural steps, and the potential release of Bisphenol-A (BPA), among other clinical scenarios that may limit their use [4–6].

An alternative to traditional RBS is the surface pre-reacted glass-ionomer (S-PRG) sealant. This material incorporates pre-reacted fluoroaluminosilicate glass particles within a resin matrix, which react with polyacrylic acid to form stable glass ionomer [7]. The material not only possesses strength comparable to that of resin composites but also continuously releases fluoride and other beneficial ions like strontium and boron [7–10]. These ions contribute to acid buffering, enamel remineralization and exhibit anti-plaque and antibacterial properties [7–10].

There are limited in vitro and clinical studies available that evaluate the retention and cariostatic efficacy of S-PRG sealants with RBS [11–13].Additionally, no study has thoroughly evaluated the effects of different pre-treatment and conditioning methods on the retention and cariostatic efficacy of S-PRG sealants. Our study aimed to further investigate the performance of S-PRG sealants under various application protocols to evaluate their penetration ability, retention, and occlusal caries inhibition. The null hypotheses tested were that (i) there is no difference in the sealant retention between RBS and S-PRG of varying pretreatments, (ii) there is no significant difference in penetration abilities between RBS and S-PRG of varying pretreatments and (iii) there is no significant difference in the potential to inhibit occlusal caries between RBS and S-PRG of varying pretreatment.

## Materials and methods

### Experimental study design

**Fig. 1:**
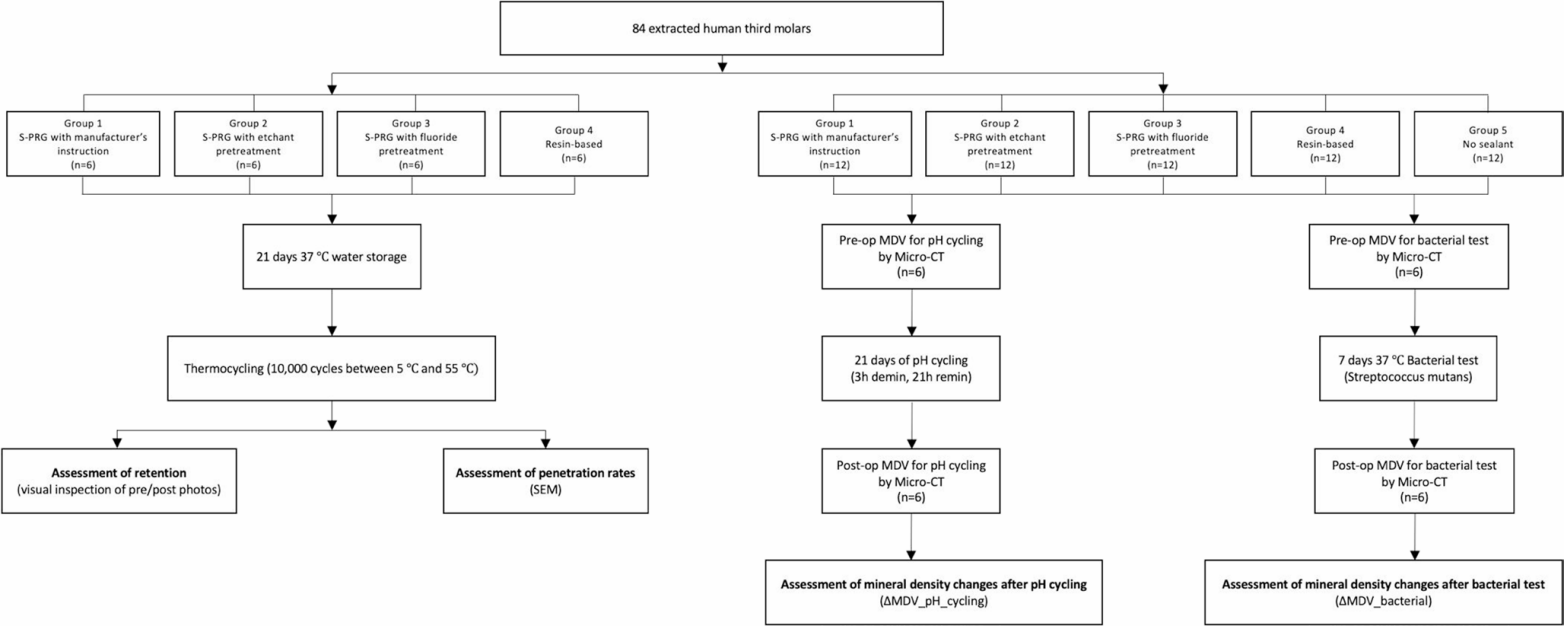
Experiment framework of the study

The experiment protocol and ethical aspects of this study were evaluated and approved by the Institutional Review Board of The University of Hong Kong–Hospital Authority Hong Kong West Cluster (Reference number: UW 20–293). An overview of the experiment framework is presented in Fig. 1, summarizing the critical phases of the methodology. In this study, tooth specimens underwent artificial aging using water storage and thermocycling, as well as exposure to cariogenic conditions simulated by pH cycling and bacterial challenge.

### Sample size calculation

Sample size estimation was performed in advance using G*Power 3.1 (Franz Faul, Germany), based on parameters from a previous study [14]. Assuming a statistical power of 0.95 and a significance level of 0.05, the minimum required sample size was calculated to be six teeth per group across five groups. Each tooth was further sectioned into three specimens for evaluation, resulting in a total of 18 specimens per group. For the assessment of fissure penetration and sealant retention, a total of 72 tooth specimens (6 tooth x 3 specimens per tooth × 4 sealant groups) were used, as a negative control group was not necessary. In contrast, the evaluation of caries-inhibitory potentials of sealants compared to no sealants through pH cycling and bacterial challenge required 90 specimens for each test to allow comparison with a no-sealant control. Therefore, a total of 84 permanent molars were used in this study, providing 252 specimens for assessment.

### Specimen preparation

Caries-free, intact, unrestored human third molars freshly extracted due to therapeutic reasons were collected from patients after obtaining their informed consent. The specimens were not aged so as to evaluate the immediate effects of sealant materials and pretreatments on caries inhibition without confounding variables from aging. After surface debridement, the teeth were carefully inspected under 8x magnification with a stereomicroscope (Carl Zeiss Stereo 475002, Oberkochen, Germany) by a dentist (first author). Teeth with caries lesions, enamel defects, including fluorosis, hypomineralization and hypoplasia, were excluded. The included teeth were subsequently immersed in 0.5% thymol solution at 37 °C until use. The roots of the teeth were removed by sectioning the tooth with a custom-made 150-µm thick saw microtome (SYD Mikki Pulley, Kawasaki, Japan) at the cementoenamel junction. Except for the occlusal surface, all other surfaces were sealed with acid-resistant nail varnish (Revlon, New York, NY, USA). The included teeth were randomly divided into 5 groups (*n* = 6) (Table 1). Three sealant application protocols were used on the S-PRG sealant (BeautiSealant, Shofu, Kyoto, Japan) groups. One etched with 37% phosphoric acid (Scotchbond™, 3 M ESPE, MN, USA) as a pretreatment before following manufacturer’s instruction, one applied with 5% NaF varnish (Duraphat, Colgate, New York, USA) 2 weeks before following manufacturer’s instruction for sealant placement, and one following manufacturer’s instruction without any additional pretreatment. One group of RBS (Helioseal, Ivoclar Schaan, Liechtenstein) served as a positive control, and a group of no sealant applied was set as a negative control. Materials were used to seal off the fissures on the occlusal surface.

**Table 1:** Sealants used and application protocols

Group	Sealant (Manufacturer)	Active component	Pretreatment conditioning	Procedure
G1	BeautiSealant (Shofu, Kyoto, Japan)	S-PRG filler (Fluoride, Sodium, Strontium, Aluminum, Silicate, Borate)	None	1. Apply BeautiSealant primer to clean teeth And leave for 5s 2. Gently air dry for 5s 3. Apply BeautiSealant directly to seal off the fissures 4. Wait for 15 s and cure with curing light (Bluephase Style, Ivoclar Vivadent, Schaan, Liechtenstein) for 20 s (light output 1100 mW/cm^2^)
G2	BeautiSealant (Shofu, Kyoto, Japan)	S-PRG filler (Fluoride, Sodium, Strontium, Aluminum, Silicate, Borate)	37% phosphoric acid etchant (Scotchbond™, 3 M ESPE, MN, USA)	1. Apply etching gel (Scotchbond™, 3 M ESPE, MN, USA) And let it react for 60s 2. Rinse and dry thoroughly and the etched enamel should have a mat white appearance 3. Apply BeautiSealant primer to clean teeth And leave for 5s 4. Gently air dry for 5s 5. Apply BeautiSealant directly to seal off the fissures 6. Wait for 15 s and cure with curing light (Bluephase Style, Ivoclar Vivadent, Schaan, Liechtenstein) for 20 s (light output 1100 mW/cm^2^)
G3	BeautiSealant (Shofu, Kyoto, Japan)	S-PRG filler (Fluoride, Sodium, Strontium, Aluminum, Silicate, Borate)	Fluoride Varnish 2 weeks ago (Duraphat, Colgate, New York, USA)	1. Fluoride varnish applied directly with microbrush And stored in deionized water at 37 °C for 2 weeks 2. Rinsed and dry thoroughly 3. Apply BeautiSealant primer to clean teeth And leave for 5s 4. Gently air dry for 5s 5. Apply BeautiSealant directly to seal off fissures 6. Wait for 15 s and cure with curing light (Bluephase Style, Ivoclar Vivadent, Schaan, Liechtenstein) for 20 s (light output 1100 mW/cm^2^)
G4	Helioseal(Ivoclar, Schaan, Liechtenstein)	Bis-GMA (bisphenol A-glycidyl methacrylate), triethylene glycol dimethacrylate		1. Apply etching gel (Scotchbond™, 3 M ESPE, MN, USA) And let it react for 60s 2. Rinse and dry thoroughly and the etched enamel should have a mat white appearance 3. Apply Helioseal directly with microbrush to seal off the fissures. 4. Wait for 15 s and cure with curing light (Bluephase Style, Ivoclar Vivadent, Schaan, Liechtenstein) for 20 s (light output 1100 mW/cm^2^)
G5	No sealant	Not applicable	None	None 5. (Negative control for pH cycling & bacterial challenge only)

### Artificial aging with water storage and thermocycling

Photographs (EOS 200D; Canon Inc., Tokyo, Japan) were taken for the four groups of specimens with sealant placement. The specimens were labelled in numbers in random sequences, followed by storage for 21 days in distilled water at 37 °C And 95% relative humidity in an incubator (Memmert Inc., Schwabach, Germany). Then, a thermocycling period of 10,000 cycles between 5 and a 55 °C with a dwell time of 20 s each was commenced [15].

### Direct visual inspection for retention

After thermocycling, the retention of sealant was first evaluated with the naked eye without any magnification. With reference to the pre-operative photographs taken at baseline, sealant retention was assessed and classified as “fully intact”, “partially intact”, or “totally loss”.

### Scanning electron microscope (SEM) for penetration

Each tooth was further sectioned in a buccolingual direction using a custom-made water-cooled diamond-impregnated low-speed saw, yielding three sections per specimen for analysis with scanning electron microscopy (SEM). The specimens were mounted on aluminum stubs, sputter-coated with platinum-palladium (IXRF Systems Inc., Austin, TX, USA) and evaluated under 45x magnification of an SEM (SU1510, Hitachi, Japan) [16].

**Fig. 2:**
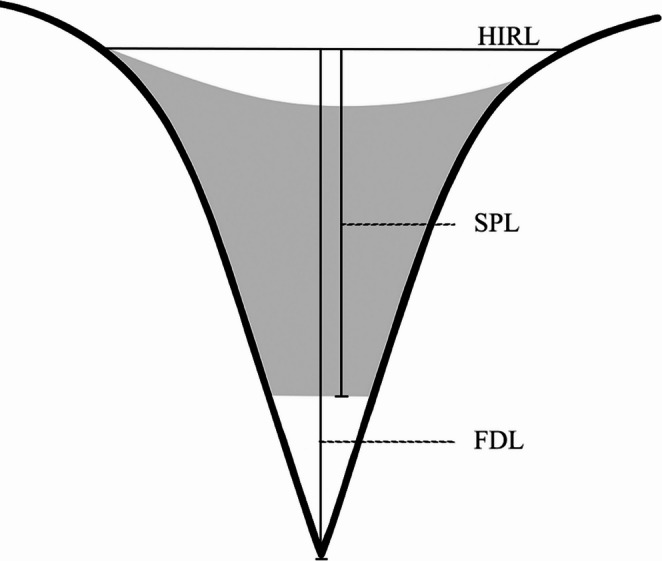
Schematic drawing of HIRL, FDL and SPL measurements

Each image, with a calibrated reference line of 500 μm drawn at the top, was used as a reference line to assess the penetration depth in relation to fissure depth. Another line, drawn below the buccal and lingual slopes perpendicular to the cusp section, served as a horizontal intercuspal reference line (HIRL). HIRL was chosen instead of the upper margin to eliminate potential bias from the uneven sealant surfaces and standardizing the assessment across varying fissure morphologies. A fissure depth line (FDL) was a line drawn from and perpendicular to the HIRL to the bottom of the fissure. Sealant penetration (SP) was measured from the lowest point where sealant was present to the HIRL on the FDL. Sealant penetration was calculated with the fraction SP/FDL and expressed in percent. Figure 2 shows the schematic drawing of HIRL, FDL and SPL measurements.

### pH cycling test

A 21-day pH cycling system [17] was used to perform the pH cycling to all specimens on an orbital shaker (Labnet, Woodbridge, NJ, USA), operated at 80-rpm at room temperature. The solutions were refreshed every 48 h to maintain ionic concentrations, and pH was monitored using the pH meter. Analytical grade chemicals (Sigma-Aldrich, St. Louis, MO, USA) and deionized water were used to prepare the buffered remineralizing and demineralizing solutions. The demineralizing solution contained 2.2mM calcium chloride dihydrate (CaCl_2_ × 2H_2_O), 1.35mM potassium dihydrogen phosphate (KH_2_PO_4_), 0.05 M acetic acid. The pH was adjusted with 5 M KOH to 4.4. The remineralizing solution consisted of 1.5 mM CaCl_2_ × 2H_2_O, 0.9 mM sodium dihydrogen phosphate (NaH_2_PO_4_), 0.15 M potassium chloride (KCl). The pH was similarly adjusted to a neutral pH of 7.0 with KOH. A state of supersaturation of apatite minerals, which simulates the condition found in saliva, was created. Each daily cycle involved one demineralization phase which lasted for three hours, alternated with one remineralization phase which lasted for twenty-one hours [18, 19]. Each specimen was immersed in 5mL of the treatment solution at each phase. Deionized water and dry fibreless laboratory-used napkins (Kimwipes™ Ex-L, Kimberly-Clark Professional, Irving, TX, USA) were used to rinse and dry the specimens between each phase.

### Streptococcus mutans bacterial test

Before bacterial exposure, all specimens were sterilized using an autoclave. Following the standardized protocols for bacterial testing [20], *Streptococcus mutans* (ATCC 35668) was initially cultured anaerobically on blood agar plates at 37 °C for 48 h. A single colony was then selected and grown anaerobically in brain heart infusion (BHI) broth (Thermo Fisher Scientific, Basingstoke, UK). Following this, bacterial cell pellets were harvested and transferred into fresh BHI broth supplemented with 5% sucrose. The bacterial suspension was standardized to a McFarland turbidity of 2 (approximately 6 × 10^8^ cells/mL). Subsequently, 1.5 mL of the standardized bacterial suspension and one tooth specimen were placed into each well of a sterile 24-well plate and incubated anaerobically at 37 °C for 7 days. After incubation, biofilms formed on the tooth specimens were detached using ultrasonic cleaning to facilitate further analysis. To maximize the sensitivity for detecting differences in sealant efficacy, a remineralization phase was not included following demineralization under cariogenic challenge in the bacterial test.

### Micro-computed tomography (Micro-CT) for mineral density assessment

Micro-CT imaging was performed (Skyscan 1276 X-Ray Microtomograph, Bruker, Kontich, Belgium) to evaluate the mineral density (MD) of tooth specimens both before and after pH cycling and bacterial testing, adhering to standard protocols [21]. Scans were conducted with a pixel size of 14 μm, and the X-ray beam was oriented parallel to the occlusal surface of each specimen, which was positioned on a computer-controlled turntable. Scanning parameters were standardized at 90 kV voltage, 200 µA current, 3.63 s integration time, and isotropic resolution of 14 μm, with a full 360° rotation in increments of 1°. Hydroxyapatite calibration disks with predetermined mineral densities (0.25 g/cm³ And 0.75 g/cm³) served as reference phantoms. Three-dimensional images were reconstructed with NRecon software (v. 1.7.0.4, SkyScan, Belgium) with uniform parameters (smoothing: 1, ring artifact correction: 2, dynamic range: 0–0.25) applied consistently at each scanning level. DataViewer software (v. 1.4.4.0, SkyScan, Belgium) facilitated precise alignment of reconstructed preoperative and postoperative images, ensuring consistent orientation. Sagittal cross-sections were selected for mineral density assessment.

Preoperative sagittal, transverse, and coronal micro-CT images were captured using DataViewer software (v.1.4.4.0, skyscan, Belgium). For each specimen, the postoperative dataset was reoriented and calibrated in the same software to match these three orthogonal views, ensuring identical alignment of anatomical axes. This registration process standardized the imaging planes and permitted consistent evaluation of corresponding regions, thereby enabling reliable comparisons of mineral density values between pre- and post-treatment scans. Measurement points were established at locations corresponding to one-third, one-half, and three-fourths along the length of each specimen. At each designated location, a cylindrical region of interest (ROI) with a volume of approximately 7.2 mm³ (based on An area of 0.6 mm² And height of 12 mm) was defined. Mean mineral density values (MDVs) were computed from micro-CT data within each ROI, resulting in three MDV measurements per specimen.

Changes in mineral density values (ΔMDV) following pH cycling and bacterial tests were compared among the five groups (*n* = 18 per group) using the Kruskal-Wallis test. ΔMDV for each specimen was calculated by subtracting the preoperative mineral density value (MDV_pre) from the postoperative mineral density value (MDV_post), both measured in g/cm³. The formula applied was ΔMDV = MDV_pre – MDV_post. Positive ΔMDV values indicate a mineral density loss, whereas negative ΔMDV values indicate an increase in mineral density following pH cycling. These calculated ΔMDV values were subsequently utilized for statistical analysis.

### Statistical analysis

Data were first organized using Microsoft Office Excel 2016 (Microsoft Office Professional Plus 2016, Microsoft, Redmond, WA, USA), then exported for statistical analysis using SPSS version 24 (IBM SPSS^®^ Statistics Inc., Chicago IL, USA). Normality of data distribution was assessed with the Shapiro-Wilk test, while homogeneity of variances was evaluated using Levene’s test. Fissure depth, sealant depth and penetration rates between 5 groups after thermocycling were evaluated with Kruskal-Wallis test followed by pairwise comparisons with Mann-Whitney U tests employing Bonferroni correction (α = 0.0083 for thermocycling tests). The changes in mineral density (ΔMDV) after pH cycling and Streptococcus mutans bacterial test were compared between groups with non-parametric analyses using the Kruskal-Wallis test, with subsequent pairwise comparisons conducted through Mann-Whitney U tests employing Bonferroni correction (α = 0.005 for pH cycling and bacterial tests). Sealant retention data were analyzed using Chi-square tests.

Mann-Whitney U tests were conducted to investigate whether sealant retention differs in penetration ability and mineral density changes. To investigate whether sealant penetration ability was associated with mineral density outcomes, Spearman’s correlation was performed. Multiple linear regression analysis was conducted to evaluate whether sealant retention and penetration could predict ΔMDV under pH cycling and bacterial test. The level of statistical significance was predetermined at α = 0.05.

## Results

### Visual inspection for sealant retention

Sealant retention was evaluated in the four experimental groups via visual inspection following a 21-day water storage period and subsequent thermocycling for 10,000 cycles. The result revealed that the S-PRG group with etchant pretreatment (G1) and the RBS (G4) each had four fully intact specimens and two partially intact specimens, indicating identical retention performance. In contrast, the S-PRG group with fluoride pretreatment (G2) and the S-PRG group applied with the manufacturer’s instruction (G3) exhibited lower retention, each with two fully intact and four partially intact specimens. No specimen showed totally loss. However, chi-square analysis indicated no statistically significant differences in sealant retention among the groups (*p* = 0.446) (Appendix 1).

### SEM for sealant penetration assessment

**Fig. 3:**
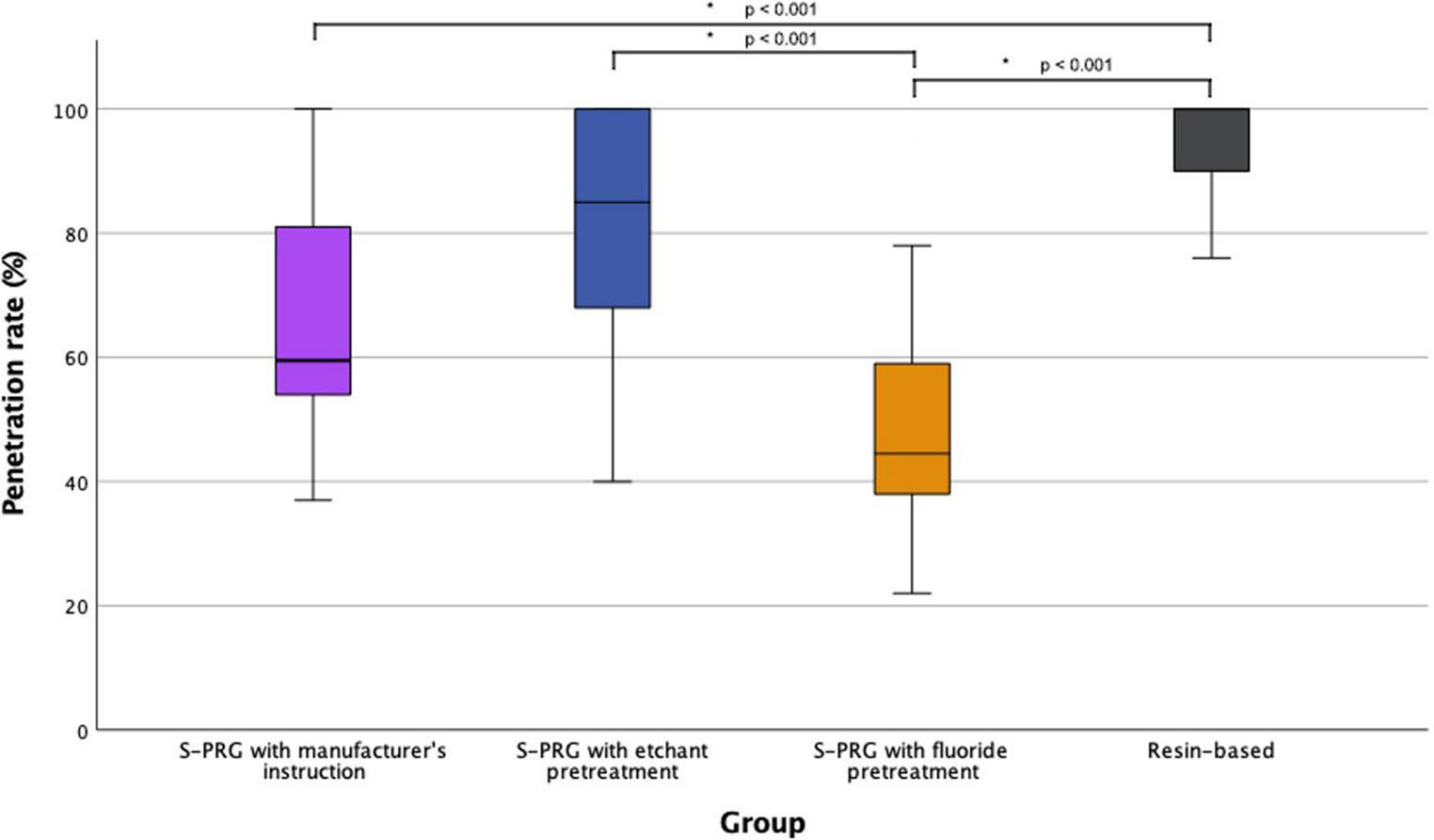
The percentage of sealant penetration rates of the four groups. The asterisk (*) indicates significant differences among the groups

Sealant penetration capability across four groups was evaluated using scanning electron microscopy (SEM). A total of eighteen measurements per group were recorded, and statistical comparisons were performed among groups. Table 2 summarizes minimum, maximum, median, interquartile range (IQR) values and associated p-values of fissure depth, sealant depth and penetration rates from each group with the pairwise comparisons of penetration rates using Mann-Whitney U tests. Figure 3 shows the box plot illustrating the penetration rates between the groups. Significant differences in fissure depth and sealant depth were observed among groups (*p* < 0.05), indicating baseline anatomical variability. These variables were not primary outcomes but may have contributed to differences observed in penetration rate.

**Table 2:** Descriptive statistics of fissure depth, sealant depth and penetration rate among the groups

Variable	Group	Minimum	Maximum	Median	IQR	Kruskal-Wallis *p* - value
Fissure Depth (µm)	G1	251	1089	714	549	*p* = 0.009
	G2	212	1066	782.5	494	
	G3	295	1087	864.5	367	
	G4	181	1008	489.5	268	
Sealant Depth (µm)	G1	185	741	447.5	170	*p* = 0.49
	G2	207	1030	553.5	376	
	G3	159	1019	368	259	
	G4	181	908	463	280	
Penetration Rate (%)	G1	37	100	59.5^ab^	30	*p* < 0.001
	G2	40	100	85^bc^	33	
	G3	22	95	44.5^a^	24	
	G4	76	100	100^c^	10	

Note: Fissure depth and sealant depth were recorded as anatomical descriptors; penetration rate was the primary outcome of interest. Superscript letters indicate statistically homogeneous subsets based on Mann–Whitney U tests with Bonferroni correction (p=0.008). Groups sharing a letter are not significantly different. G1: Surface pre-reacted glass (S-PRG) sealant applied per manufacturer's instructions; G2: S-PRG with 37% phosphoric acid etching pretreatment; G3: S-PRG with fluoride varnish pretreatment; G4: Resin-based sealant (RBS); G5: No-sealant control

Statistically significant differences were identified between the S-PRG with manufacturer’s instruction group (G1) and the RBS group (G4) (*p* < 0.001), between the S-PRG with fluoride pretreatment group (G3) and the RBS group (G4) (*p* < 0.001), and between the S-PRG with etchant pretreatment group (G2) and the S-PRG with fluoride pretreatment group (G3) (*p* < 0.001). No significant differences emerged from other pairwise comparisons. Sealant penetration rankings based on median percentages and IQR indicated the RBS group (G4) had the highest penetration (100 ± 10%), followed by the S-PRG with etchant pretreatment group (85.0 ± 33%), S-PRG with manufacturer’s instruction group (59.5 ± 30%), and S-PRG with fluoride pretreatment group (44.5 ± 24%), respectively (Appendix 2).

### pH cycling: mineral density changes (ΔMDV)

**Fig. 4:**
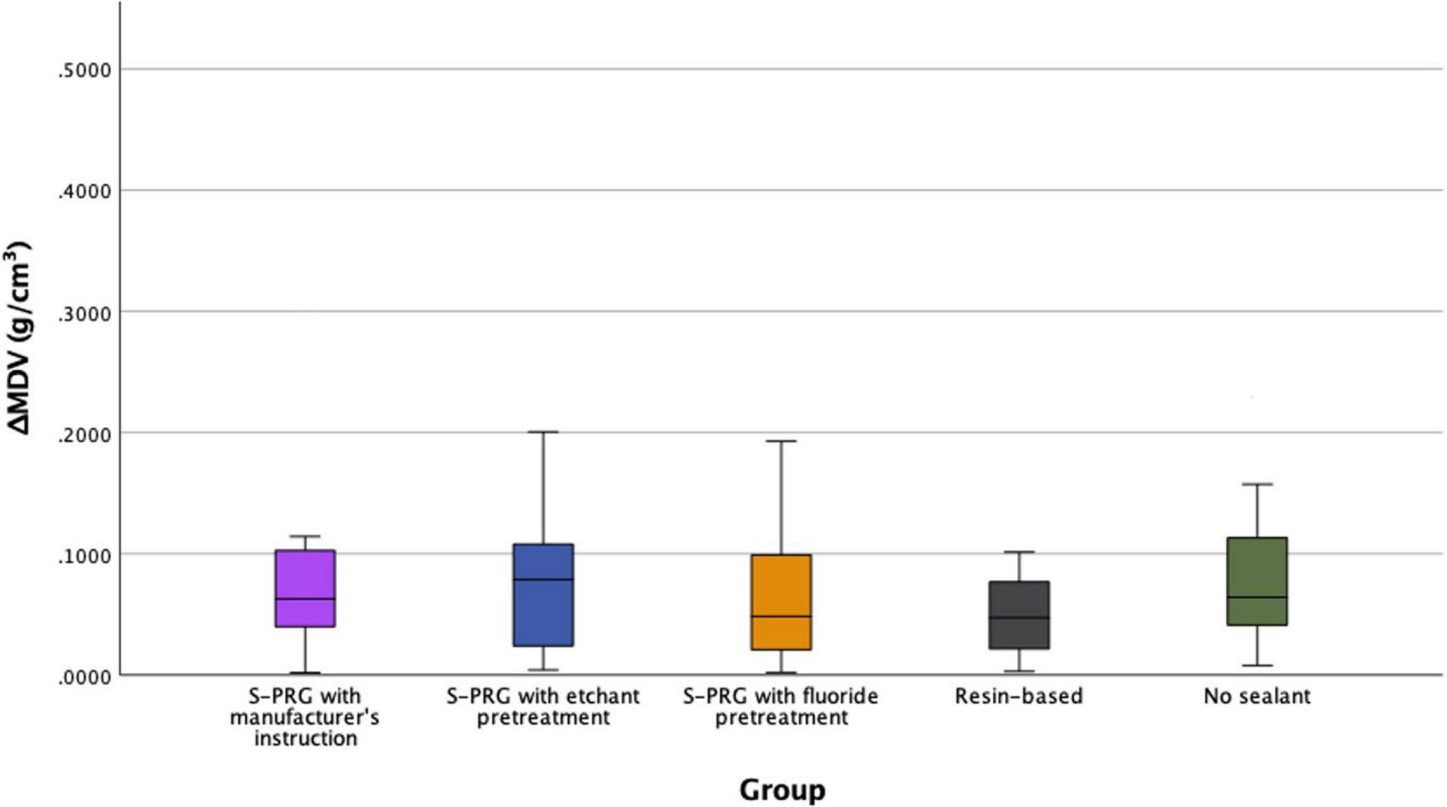
Mineral density changes (ΔMDV) of pH cycling test. The asterisk (*) indicates significant differences among the groups

The Kruskal-Wallis analysis revealed no statistically significant differences in ΔMDV values among groups (H = 3.717, df = 4, *p* = 0.446), indicating that the type of sealant and its application method did not significantly affect the mineral density changes under pH cycling test. Table 3 shows the minimum, maximum, median and interquartile range (IQR) values of ΔMDV and p-value of each group. Figure 4 shows the boxplot illustrating the mineral density changes between groups.

**Table 3:** Descriptive statistics of mineral density change (ΔMDV) (g/cm³) of pH cycling among the groups

Group	Sealant Type	Minimum ΔMDV (g/cm³)	Maximum ΔMDV (g/cm³)	Median ΔMDV (g/cm³)	IQR	Kruskal-Wallis *p* - value
G1	S-PRG sealant with manufacturer’s instruction	0.0020	0.3054	0.0630	0.0688	*p* = 0.446
G2	S-PRG sealant with etchant pretreatment	0.0042	0.2551	0.0790	0.0943	
G3	S-PRG sealant with fluoride pretreatment	0.0021	0.2283	0.0484	0.0861	
G4	Resin-based sealant	0.0031	0.1015	0.0473	0.0590	
G5	No sealant	0.0080	0.3444	0.0643	0.0852	

Note: No statistically significant differences were found among the based on Mann–Whitney U tests with Bonferroni correction (p$\:>$0.0083). Superscript letters were not applied

G1: Surface pre-reacted glass (S-PRG) sealant applied per manufacturer’s instructions

G2: S-PRG with 37% phosphoric acid etching pretreatment

G3: S-PRG with fluoride varnish pretreatment

G4: Resin-based sealant (RBS) (G4)

G5: No-sealant control

### S. mutans bacterial test: mineral density changes (ΔMDV)

**Fig. 5:**
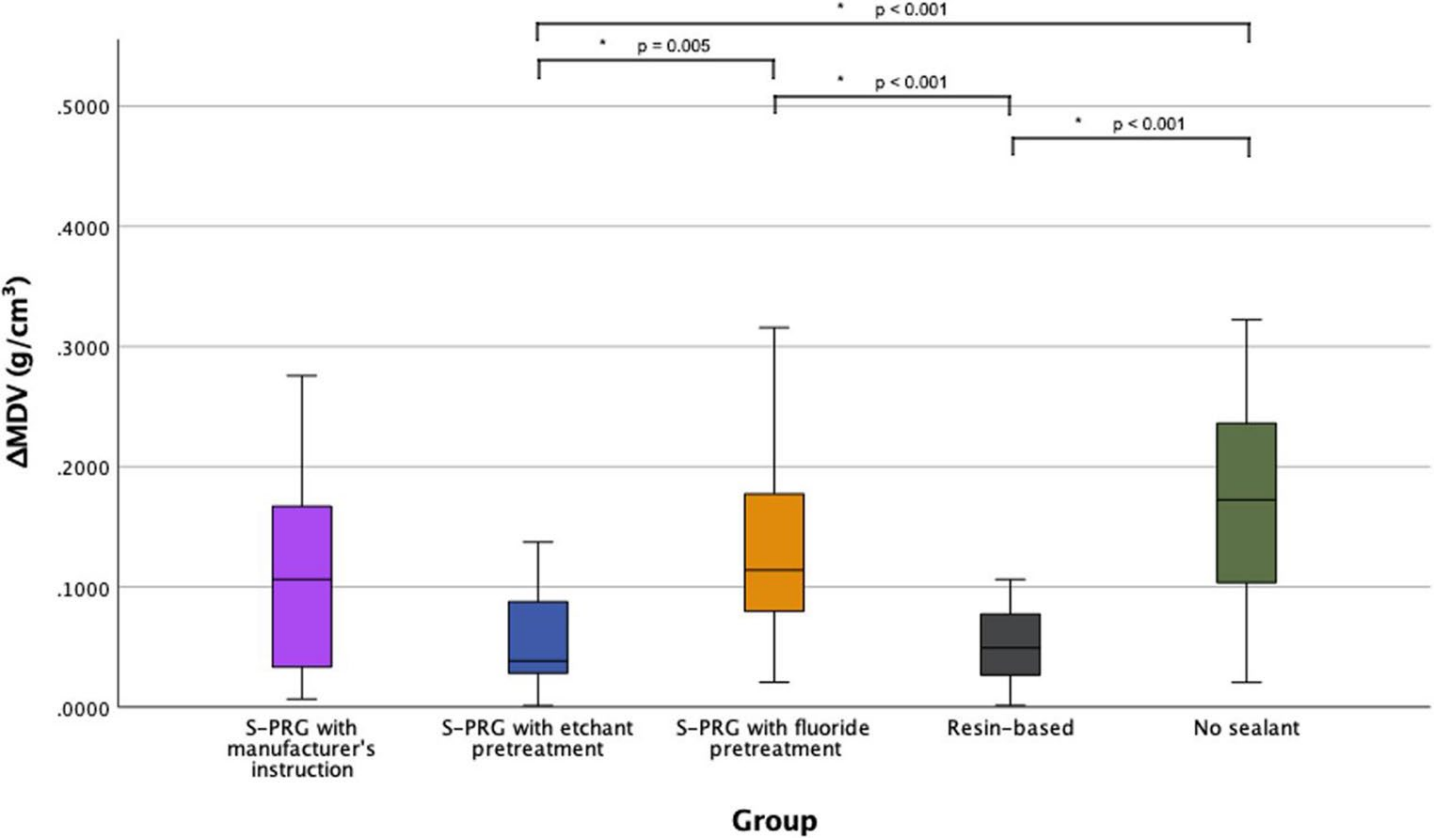
Mineral Density Changes (ΔMDV) of bacterial test. The asterisk (*) indicates significant differences among the groups

Table 4 shows the minimum, maximum, median and IQR values of ΔMDV and p-value of each group with the pairwise comparisons of ΔMDV using Mann-Whitney U tests. Figure 5 shows the box plot illustrating the mineral density changes between groups. The Kruskal-Wallis test revealed significant differences among the five groups (*p* < 0.001). Mann-Whitney U test with Bonferroni correction (α = 0.005) showed a significant difference in pairwise comparisons. The S-PRG with etchant pretreatment group (G2) demonstrated the lowest median ΔMDV (0.0385, IQR: 0.0635), followed by the RBS group (G4), which had a median ΔMDV of 0.0492 (IQR: 0.0515). Both groups showed statistically significant differences when compared to the S-PRG with fluoride pretreatment group (G3) (G2 vs. G3: *p* = 0.005; G4 vs. G3: *p* < 0.001) and the group without sealant application (G5) (G2 vs. G5: *p* < 0.001; G4 vs. G5: *p* < 0.001). No other pairwise comparisons revealed statistically significant differences.

**Table 4:** Descriptive statistics of mineral density change (ΔMDV) (g/cm³) of bacterial test among the groups

Group	Sealant Type	Minimum ΔMDV (g/cm³)	Maximum ΔMDV (g/cm³)	Median ΔMDV (g/cm³)	IQR	Kruskal-Wallis *p* - value
G1	S-PRG sealant with manufacturer’s instruction	0.0067	0.2757	0.1062^abcd^	0.1476	*p* < 0.001
G2	S-PRG sealant with etchant pretreatment	0.0012	0.2107	0.0385^ad^	0.0635	
G3	S-PRG sealant with fluoride pretreatment	0.0208	0.3158	0.1140^bc^	0.0992	
G4	Resin-based sealant	0.0015	0.1064	0.0492^ad^	0.0515	
G5	No sealant	0.0206	0.5330	0.1723^bc^	0.1498	

Note: Superscript letters indicate statistically homogeneous subsets based on Mann–Whitney U tests with Bonferroni correction (p ≤ 0.005). Groups sharing a letter are not significantly different.

G1: Surface pre-reacted glass (S-PRG) sealant applied per manufacturer’s instructions

G2: S-PRG with 37% phosphoric acid etching pretreatment

G3: S-PRG with fluoride varnish pretreatment

G4: Resin-based sealant (RBS) (G4)

G5: No-sealant control

### Effects of sealant retention and penetration ability on mineral density under conditions of pH cycling and bacterial test

The Mann-Whitney U tests showed no significant differences in penetration ability (*p* = 0.349), mineral density changes under pH cycling (*p* = 0.065) or bacterial test (*p* = 0.564) among different sealant retention types. Spearman’s rank correlation revealed a statistically significant negative association between penetration percentage and mineral density changes under bacterial challenge (ρ = − 0.244, *p* = 0.039), while no significant correlation was found under pH cycling conditions (*p* = 0.289). Multiple linear regression analysis showed that the model was statistically significant (R² = 0.313, *p* = 0.019), indicating that 31.3% of the variance in mineral density change (ΔMDV) under bacterial challenge was explained by the predictors. Penetration percentage was a significant predictor (*p* = 0.006), with higher penetration associated with lower mineral loss. Retention status, however, did not significantly contribute to the model (*p* = 0.308). Additionally, sealant retention and penetration were not a significant predictor of ΔMDV in the pH cycling model (R² = 0.225, *p* = 0.068).

## Discussion

This study considered both types of sealant materials and the surface pretreatment methods to assess their impact on sealant retention, fissure penetration, and occlusal caries inhibition. Based on the results, the first null hypothesis, which stated that there would be no significant difference in sealant retention among groups, could not be rejected, as no statistically significant differences were observed. However, the second null hypothesis, related to fissure penetration, and the third null hypothesis, concerning occlusal caries inhibition, were both rejected due to the statistically significant differences identified between specific groups.

No statistically significant differences in sealant retention were observed among the groups following artificial aging. However, the S-PRG sealant applied according to the manufacturer’s instruction exhibited greater retention loss compared to the RBS group. This trend aligns with findings from Penha et al., who reported lower retention rates for S-PRG sealants after 12 months of clinical evaluation [12]. In addition, another clinical study demonstrated that the use of phosphoric acid etchant pretreatment significantly improved the retention of S-PRG sealants [22]. Consistent with these findings, our in vitro study observed a higher number of fully intact sealants in the S-PRG group with phosphoric acid pretreatment compared to the non-etched S-PRG groups. Although the differences in retention were not statistically significant in our study, the collective evidence from these clinical studies, alongside our lab findings, suggests that pretreatment with phosphoric acid may enhance the retention performance of S-PRG sealants. The absence of significant retention differences here likely arises from the controlled in vitro conditions, specifically the lack of dynamic masticatory forces, saliva exposure, and enzymatic degradation, factors significantly impacting sealant longevity clinically.

In evaluating penetration ability, the RBS demonstrated the highest penetration percentage, consistent with its inherently lower viscosity and superior flow properties [16]. To the best of our knowledge, few papers have investigated the viscosity of S-PRG sealants. Given their incorporation of multiple ion-releasing glass fillers, it is reasonable to hypothesize that S-PRG sealants exhibit higher viscosity than conventional resin-based sealants. Although both materials demand careful moisture control per manufacturers’ instructions, lower-viscosity sealants may achieve more consistent infiltration into etched enamel fissures when slight moisture contamination is present. Future studies should directly measure sealant rheology and investigate its impact on penetration and retention under simulated oral humidity. Notably, S-PRG sealant with 37% phosphoric acid etching pretreatment (G2) showed enhanced penetration ability compared to the manufacturer’s instruction group (G1) and fluoride pretreatment group (G3) which only self-etch technique was used, although differences between Groups 1 And 2 were not statistically significant. The reduced effectiveness of the self-etch technique has been attributed to the relatively low acidity of self-etch adhesives, which are insufficient to adequately etch uncut enamel. This results in incomplete resin infiltration and limited resin tag formation, ultimately compromising the sealant’s bond strength. The purpose of the etching step is to remove the smear layer, selectively dissolve enamel rods, and create macro- and microporosities that facilitate resin adhesion. Following etching, hydrophobic resin penetrates the conditioned enamel via capillary action, and upon light curing, establishes micromechanical interlocking essential for durable bonding and deeper sealant infiltration [12, 22, 23].

The lowest penetration percentage observed in the fluoride pretreatment group may be attributed to interference from residual fluoride varnish, which likely impeded the flow of sealant into the fissures. The inherent tackiness of the fluoride varnish, due to its resinous carrier, left a tenacious, adhesive film on enamel. This obstruction could have negatively affected sealant infiltration and contributed to the reduced penetration outcome. Supporting this interpretation, SEM images revealed the presence of a distinct intervening layer between the enamel surface and the sealant in the fluoride group, a feature not observed in the SEM images of the other groups.

For mineral density changes assessed via pH cycling, the lack of statistically significant differences in the pH cycling test suggests that all sealant groups provided comparable resistance to mineral loss under chemically induced demineralization–remineralization conditions. In contrast, significant differences emerged under the *Streptococcus mutans* bacterial challenge, indicating that the sealants’ performance varied more noticeably when subjected to a biologically driven cariogenic environment. This discrepancy may be explained by the added complexity of bacterial acid production, biofilm formation, and microbial adherence, which creates a more aggressive and dynamic demineralization process than the chemically controlled pH cycling model. As such, the bacterial test may better reflect clinical conditions, particularly the bio-interactive properties of the sealant.

S-PRG sealant with 37% phosphoric acid etching pretreatment (G2) exhibited the lowest mineral density loss, indicating superior protection, closely followed by the RBS (G4). This superior performance in bacterial conditions may be due to the dual effect of enhanced mechanical sealing from acid pretreatment and bioactive ions release from S-PRG materials, both contributing to localized remineralization and resistance to bacterial acid production. Similar findings have been reported in previous laboratory studies, which demonstrated that S-PRG sealants possess ion-releasing properties that contribute to resistance against demineralization and enhance their capacity for caries inhibition [19, 24, 25]. However, in contrast to the findings of the present study, a previous investigation utilizing *S. mutans* as the bacterial challenge reported that S-PRG sealant exhibited weaker antibacterial properties and greater biofilm formation compared to a fluoride-releasing RBS [26]. This contrasts with our results, in which both the S-PRG sealants and RBS demonstrated comparable and favorable caries-preventive effects [26]. However, since this study did not specifically evaluate the antibacterial effects based on bacterial counts and biofilm formation between sealants, further research is needed to investigate and compare the antibacterial properties of different sealants.

Correlation analysis revealed a significant negative relationship between sealant penetration and mineral density change under bacterial conditions, indicating that better penetration may help minimize mineral loss under microbial challenge. Multiple regression supported this finding, revealing that sealant penetration significantly predicted mineral density changes in bacterial tests, accounting for approximately 10.2% of the variance. Although it is well established that multiple factors such as marginal adaptation, fissure morphology, and sealant composition, contribute to the caries-preventive efficacy of sealants [27–29], the present findings nonetheless underscore the critical role of optimizing sealant penetration during application to enhance protective outcomes.

Interestingly, no significant correlation was found between penetration ability and mineral density changes under pH cycling conditions. This discrepancy may originate from the milder nature of pH cycling, where chemical remineralization dynamics predominate rather than aggressive bacterial acid production. It is possible that sealant penetration depth becomes more crucial under the harsher, biologically driven acidic conditions.

The lack of correlation between retention rates and mineral density outcomes was found in our study. This in vitro finding is consistent with clinical studies on S-PRG sealants, which have reported that despite reduced long-term retention, caries incidence remained low, likely due to the material’s sustained ion release and bioactive effects [11, 30]. This convergence suggests that caries inhibition is not solely dependent on mechanical retention, but also on the material’s capacity to promote remineralization and maintain marginal integrity.

Clinically, the findings suggest phosphoric acid pretreatment could enhance sealant efficacy in terms of penetration and caries inhibition, providing clinicians with a valuable strategy for managing high-risk patients or challenging clinical scenarios. RBS remains an effective choice where moisture control and patient cooperation permit, but S-PRG sealants, especially when combined with acid etching, represent a compelling alternative given their bioactive potential. There were some limitations in the present study. This in vitro study was performed under laboratory conditions, which do not fully replicate the complex and dynamic environment of the oral cavity. Important clinical variables such as salivary flow, masticatory forces, and patient-related factors were not simulated, potentially limiting the generalizability of the findings to in vivo settings. Additionally, samples in the S-PRG sealant with fluoride pretreatment group were stored in deionized water rather than artificial saliva. Distilled water was chosen in order to standardize the aging environment and isolate the effects of hydrolytic degradation on sealant materials. However, distilled water may not accurately mimic oral conditions and could affect fluoride interaction. Mechanical loading was not incorporated into the thermocycling process to establish baseline performance under controlled conditions. Future research could consider combining thermocycling with mechanical loading to better simulate the masticatory forces experienced in the oral environment. Fluoride and other ion release was not measure in our study which is a critical mechanism underlying S-PRG caries-inhibition effects. S-PRG sealants continuously release fluoride, strontium, and borate ions under acidic challenges. Fluoride promotes fluorapatite formation and buffers demineralization cycles, while strontium and borate enhance acid resistance and exert antimicrobial effects against *S. mutans*. The greater penetration achieved in the phosphoric-etched group (G2) likely increased the interfacial surface area for ion diffusion into enamel fissures, thereby augmenting the sealant’s therapeutic efficacy compared with the unetched group (G3). While fissure and sealant depths were recorded, fissure anatomical variations among teeth may have influenced sealant penetration, introducing potential confounding effects. Furthermore, the long-term behavior of the materials, including degradation and wear, was not evaluated and warrants further investigation. Future research should focus on long-term clinical trials assessing the performance of S-PRG sealants with various pretreatments under actual oral conditions, integrating clinical outcomes, patient-centered factors, and material durability assessments to provide comprehensive recommendations.

## Conclusion

Within the limitation of this in vitro study, the findings can be concluded as:

1. 37% phosphoric acid pretreatment may enhance the penetration ability of S-PRG sealants compared to application following the manufacturer’s instruction alone.
2. S-PRG sealant with etchant pretreatment demonstrated caries inhibition efficacy comparable to that of RBS, with both outperforming S-PRG sealant following fluoride pretreatment.
3. In the context of S-PRG sealants, penetration depth may contribute to caries prevention, whereas retention rated does not appear to be a determining factor.
4. Penetration rate significantly predicts mineral density change under bacterial challenge, with deeper infiltration associated with less mineral loss.

## Declaration of generative AI and AI-assisted technologies in the writing process

During the preparation of this work the authors used ChatGPT(version3.5) to improve the language and readability of the manuscript. After using this tool, the authors reviewed and edited the content as needed and take full responsibility for the content of the published article.

## Supplementary Information

Below is the link to the electronic supplementary material.

ESM 1 (PDF 390 KB)

## Data Availability

The data that support the findings of this study are available from the corresponding author, C.K.Y.Y., upon reasonable request.

## References

[1] Welbury R, Raadal M, Lygidakis N (2004) EAPD guidelines for the use of pit and fissure sealants. Eur J Paediatr Dent 5:179–184 15471528

[2] Wright JT, Crall JJ, Fontana M, Gillette EJ, Novy BB, Dhar V, Donly K, Hewlett ER, Quinonez RB, Chaffin J, Crespin M, Iafolla T, Siegal MD, Tampi MP, Graham L, Estrich C, Carrasco-Labra A (2016) Evidence-based clinical practice guideline for the use of pit-and-fissure sealants: A report of the American dental association and the American academy of pediatric dentistry. J Am Dent Assoc 147(e12):672–682. 10.1016/j.adaj.2016.06.001 27470525 10.1016/j.adaj.2016.06.001

[3] Lam PP, Sardana D, Lo EC, Yiu CK, EVIDENCE-BASED META, -EVALUATION OF SEALANTS’ EFFECTIVENESS IN CARIES PREVENTION AND ARREST (2021) J Evid Based Dent Pract 21:101587. doi: 10.1016/j.jebdp.2021.101587 10.1016/j.jebdp.2021.101587 34479663

[4] Naaman R, El-Housseiny AA, Alamoudi N (2017) The use of pit and fissure sealants-a literature review. Dent J (Basel) 5. 10.3390/dj5040034 10.3390/dj5040034 PMC5806970 29563440

[5] Kantovitz KR, Pascon FM, Alonso RC, Nobre-dos-Santos M, Rontani RM (2008) Marginal adaptation of pit and fissure sealants after thermal and chemical stress. A SEM study. Am J Dent 21:377–382 19146131

[6] Mehrabkhani M, Mazhari F, Sadeghi S, Ebrahimi M (2015) Effects of sealant, viscosity, and bonding agents on microleakage of fissure sealants: an in vitro study. Eur J Dent 9:558–563. 10.4103/1305-7456.172631 26929696 10.4103/1305-7456.172631 PMC4745239

[7] Ogawa Y, Sayed M, Hiraishi N, Al-Haj Husain N, Tagami J, Özcan M, Shimada Y (2022a) Effect of surface Pre-Reacted glass ionomer containing dental sealant on the Inhibition of enamel demineralization. J Funct Biomaterials 13. 10.3390/jfb13040189 10.3390/jfb13040189 PMC9624343 36278658

[8] Shimazu K, Ogata K, Karibe H (2011) Evaluation of the ion-releasing and recharging abilities of a resin-based fissure sealant containing S-PRG filler. Dent Mater J 30:923–927 22123018 10.4012/dmj.2011-124

[9] Thuy TT, Nakagaki H, Kato K, Hung PA, Inukai J, Tsuboi S, Nakagaki H, Hirose MN, Igarashi S, Robinson C (2008) Effect of strontium in combination with fluoride on enamel remineralization in vitro. Arch Oral Biol 53:1017–1022. 10.1016/j.archoralbio.2008.06.005 18672228 10.1016/j.archoralbio.2008.06.005

[10] Han L (2007) Evaluation of selected properties of a prototype S-PRG filler containing root canal sealer. Jpn J Conserv Dent 50:713–720

[11] Ntaoutidou S, Arhakis A, Tolidis K, Kotsanos N (2018) Clinical evaluation of a surface pre-reacted glass (S-PRG) filler-containing dental sealant placed with a self-etching primer/adhesive. Eur Arch Paediatr Dent 19:431–437. 10.1007/s40368-018-0379-z 30328064 10.1007/s40368-018-0379-z

[12] Penha KJS, Roma F, Filho EMM, Ribeiro CCC, Firoozmand LM (2021) Bioactive self-etching sealant on newly erupted molars: A split-mouth clinical trial. J Dent 115:103857. 10.1016/j.jdent.2021.103857 34699954 10.1016/j.jdent.2021.103857

[13] Ataol E, Ertan A, Cehreli Z (2016) Sealing effectiveness of fissure sealants bonded with universal adhesive systems: influence of different etching modes. J Adhes Sci Technol 31:1–9. 10.1080/01694243.2016.1268378

[14] Kramer N, Schmidt M, Lucker S, Domann E, Frankenberger R (2018) Glass ionomer cement inhibits secondary caries in an in vitro biofilm model. Clin Oral Investig 22:1019–1031. 10.1007/s00784-017-2184-1 28741172 10.1007/s00784-017-2184-1

[15] AlQahtani A, Al-Dlaigan Y, Almahdy A (2022) Microtensile bond strength of bioactive pit and fissure sealants bonded to primary and permanent teeth. Materials 15:1369. 10.3390/ma15041369 35207906 10.3390/ma15041369 PMC8875102

[16] Markovic D, Petrovic B, Peric T, Miletic I, Andjelkovic S (2011) The impact of fissure depth and enamel conditioning protocols on glass-ionomer and resin-based fissure sealant penetration. J Adhes Dent 13:171–178. 10.3290/j.jad.a19006 21594230 10.3290/j.jad.a19006

[17] Yu OY, Mei ML, Zhao IS, Li QL, Lo EC, Chu CH (2018) Remineralisation of enamel with silver diamine fluoride and sodium fluoride. Dent Mater 34:e344–e352. 10.1016/j.dental.2018.10.007 30482611 10.1016/j.dental.2018.10.007

[18] Arnaud TMS, de Barros Neto B, Diniz FB (2010) Chitosan effect on dental enamel de-remineralization: an in vitro evaluation. J Dent 38:848–852 20600551 10.1016/j.jdent.2010.06.004

[19] Ogawa Y, Sayed M, Hiraishi N, Al-Haj Husain N, Tagami J, Özcan M, Shimada Y (2022) Effect of surface pre-reacted glass ionomer containing dental sealant on the inhibition of enamel demineralization. J Funct Biomater 13:189 36278658 10.3390/jfb13040189 PMC9624343

[20] Bijle MN, Abdalla MM, Ashraf U, Ekambaram M, Yiu CKY (2021) Enamel remineralization potential of arginine-fluoride varnish in a multi-species bacterial pH-cycling model. J Dent 104:103528. 10.1016/j.jdent.2020.103528 10.1016/j.jdent.2020.103528 33188848

[21] Bijle MNA, Ekambaram M, Lo EC, Yiu CKY (2018) The combined enamel remineralization potential of arginine and fluoride toothpaste. J Dent 76:75–82. 10.1016/j.jdent.2018.06.009 29935996 10.1016/j.jdent.2018.06.009

[22] Elmokanen MA, Gad HMA (2024) Retention rate of Giomer S-PRG filler containing pit and fissure sealant applied with or without etching: a randomized clinical trial. BMC Oral Health 24:1356 39511504 10.1186/s12903-024-05096-7 PMC11546273

[23] Althomali YM, Musa S, Manan NM, Nor NAM (2022) Retention evaluation of fissure sealants applied using self-etch and conventional acid-etch techniques: a randomized control trial among schoolchildren. Pediatr Dent 44:249–254 35999682

[24] Nomura R, Morita Y, Matayoshi S, Nakano K (2018) Inhibitory effect of surface pre-reacted glass-ionomer (S-PRG) eluate against adhesion and colonization by Streptococcus mutans. Sci Rep 8:5056 29568011 10.1038/s41598-018-23354-x PMC5864963

[25] Kaga M, Kakuda S, Ida Y, Toshima H, Hashimoto M, Endo K, Sano H (2014) Inhibition of enamel demineralization by buffering effect of S-PRG filler‐containing dental sealant. Eur J Oral Sci 122:78–83 24372898 10.1111/eos.12107

[26] An J, Park H, Seo H, Lee S (2015) Antibacterial properties of pit and fissure sealant containing S-PRG filler on *Streptococcus mutans*. J Korean Acad Pediatr Dent 42:302–311

[27] Garg N, Indushekar K, Saraf BG, Sheoran N, Sardana D (2018) Comparative evaluation of penetration ability of three pit and fissure sealants and their relationship with fissure patterns. J Dent 19:92 PMC5960740 29854882

[28] Selecman JB, Owens BM, Johnson WW (2007) Effect of preparation technique, fissure morphology, and material characteristics on the in vitro margin permeability and penetrability of pit and fissure sealants. Pediatr Dent 29:308–314 17867396

[29] Khogli AE, Cauwels R, Vercruysse C, Verbeeck R, Martens L (2013) Microleakage and penetration of a hydrophilic sealant and a conventional resin-based sealant as a function of preparation techniques: a laboratory study. Int J Paediatr Dent 23:13–22 22276649 10.1111/j.1365-263X.2011.01218.x

[30] de Souza Penha KJ, de Oliveira Roma FRV, Dos Santos MJ, do Couto GAS, Firoozmand LM (2022) In vitro and in vivo performance of self-conditioning sealants with pre-reacted glass for caries prevention. J Mech Behav Biomed Mater 133:105304 35688036 10.1016/j.jmbbm.2022.105304

