# Supplementary material for: Efficacy of sealants with surface pre-reacted glass (S-PRG) in retention, fissure penetration and occlusal caries Inhibition: Short title: efficacy of S-PRG sealants
Source: Clin Oral Investig. 2025 Oct 13;29(11):506. doi: 10.1007/s00784-025-06551-7 (PMC12518482; doi:10.1007/s00784-025-06551-7)
Supplement: Supplementary file 1 — (PDF 390 KB) [file 784_2025_6551_MOESM1_ESM.pdf]

Appendix 1. Visual inspection for sealant retention

Groups	G1	G2	G3	G4
Total retention	5	5	4	6
Partial retention	1	1	2	0

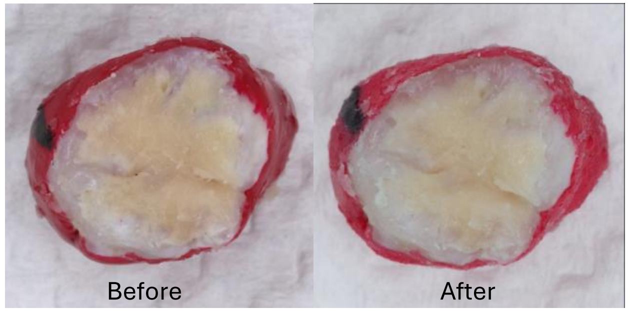

Group 1: High viscosity GI sealant (Ketac Molar)

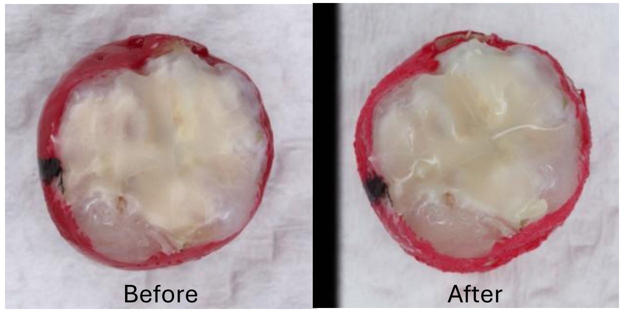

Group 2: Medium viscosity GI sealant (Fuji VII)

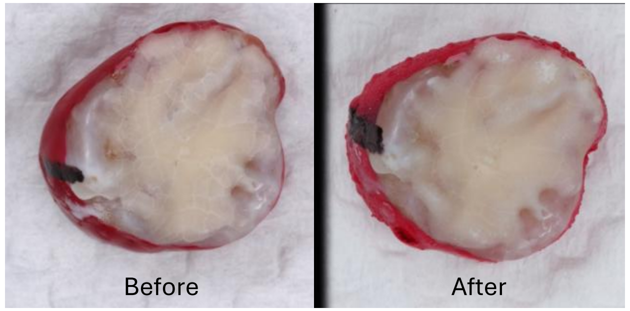

Group 3: Low viscosity GI sealant (Fuji TRIAGE)

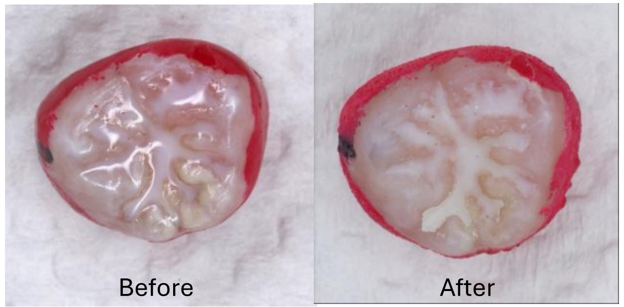

Group 4: Resin-based sealant (Helioseal)

Sealant Retention

(Hassan et al., 2019)

Category 1	Total retention
Category 2	Partial retention
Category 3	Total loss

# Appendix 2. SEM for sealant penetration assessment

Group	G1	G2	G3	G4
Median (%)	59.5	85	44.5	100

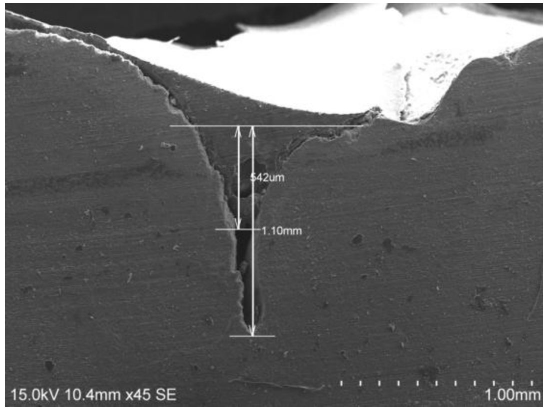

Group 1: S-PRG sealant (manufacturer's instruction)

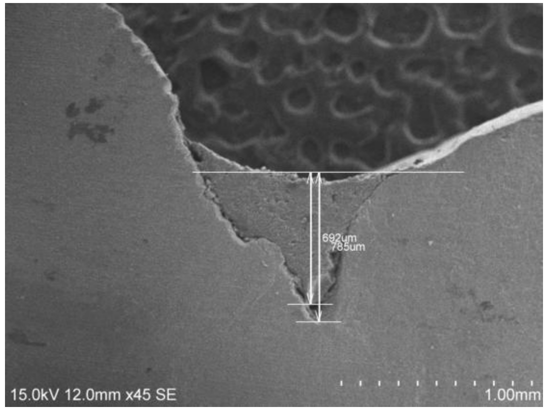

Group 2: S-PRG sealant (37% phosphoric acid)

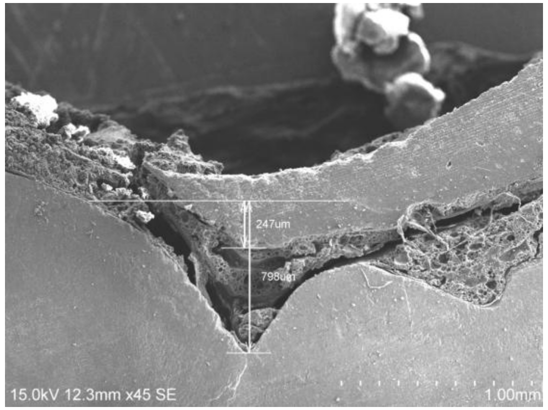

Group 3: S-PRG sealant (Fluoride varnish 2 weeks ago)

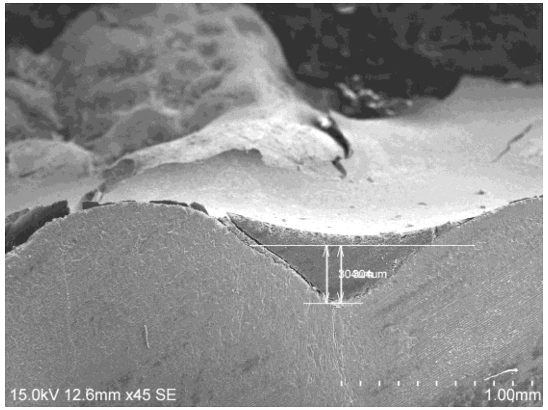

Group 4: Resin-based sealant (Helioseal)
